# Clinical characteristics of fatal cases of hand, foot and mouth disease in children

**DOI:** 10.3389/fped.2025.1522164

**Published:** 2025-07-17

**Authors:** Xue Zhang, Ruiyang Sun, Jiapu Hou, Wanyu Jia, Peng Li, Shengli Shi, Chunlan Song, Yibing Cheng

**Affiliations:** Henan Province Engineering Research Center of Diagnosis and Treatment of Pediatric Infection and Critical Care, Children’s Hospital Affiliated to Zhengzhou University, Henan, China

**Keywords:** hand, foot and mouth disease, fatal cases, enterovirus A71, death cause, clinical characteristics

## Abstract

**Background:**

Hand, foot and mouth disease (HFMD) is a common infectious disease that continues to pose a serious threat to children. The purpose of this study was to analyze the clinical characteristics and causes of death of mortality among children with fatal HFMD.

**Methods:**

Clinical data from children who had HFMD and died in the pediatric intensive care units of Henan Children's Hospital from January 2014 to December 2019 were retrospectively analyzed.

**Results:**

Thirty-one fatal cases were identified (19 males, 12 females), aged 9–47 months, all without underlying disease. Mortality was highest among children aged 12–36 months. A declining trend in deaths was observed from 2014 to 2019. The median duration of disease was 4.5 days. Common clinical manifestations included fever, rash, dyspnea, disturbance of consciousness, abnormal heart rate and limb tremors. Neurogenic pulmonary edema (NPE) was the primary cause of death in 24 cases, followed by brainstem encephalitis in 6 cases and cerebral hernia in 1 case. Enterovirus A71 (EV-A71) was the predominant pathogen (27 cases, 87.10%).

**Conclusion:**

Although the HFMD mortality has shown a downward trend in recent years, children younger than 36 months infected with EV-A71 remain at high risk of fatal outcomes. NPE and brainstem encephalitis are the leading causes of death in children with HFMD.

## 1 Introduction

Hand, foot and mouth disease (HFMD) is an acute infectious disease caused by enterovirus infection, with high incidence and high infectivity (1–3). HFMD is more common in children, especially in children under 5 years of age (4). According to reports by the Chinese National Center for Disease Control and Prevention (https://www.chinacdc.cn), the annual incidence and mortality rates of HFMD in China has been among the top three class C infectious diseases from 2014 to 2019. HFMD has become a major threat to public health and a considerable economic burden.

Coxsackievirus A16 (CV-A16) and enterovirus A71 (EV-A71) represent the predominant pathogens responsible for HFMD. However, the prevalence of other pathogens, such as coxsackievirus A6 (CV-A6) and coxsackievirus A10 (CV-A10), is increasingly recognized (5–7). HFMD is mostly mild and self-limiting, presenting with fever, macular papules or blisters on hands, feet and buttocks, and ulcers in the oral mucosa (8). In severe cases, children with HFMD may develop neurological, respiratory, and circulatory systems, precipitating rapid clinical deterioration. Critical complications include neurogenic pulmonary edema (NPE), aseptic meningitis, brainstem encephalitis, myocarditis and cardiopulmonary failure (4). These complications are associated with accelerated disease progression and significantly elevated mortality. NPE is the leading cause of death among children with HFMD, with most fatalities occurring within 12–18 h of symptom onset. Persistent neurological sequelae are frequently observed among survivors (9, 10).

As a major infectious public health problem in China, the prevention and control system of HFMD still faces severe challenges. At present, no specific antiviral treatment plan has been established for enterovirus infections (11, 12). Although the EV-A71 inactivated vaccine has been marketed in China and included in the immunization program, limited by the inherent high variability of RNA viruses and the lack of cross-protective effects of the EV-A71 vaccine against other serotypes of enteroviruses, the existing vaccines are difficult to achieve long-term broad-spectrum immune defense (13). Therefore, developing multivalent combination vaccines to cover the main prevalent strains and designing new antiviral drugs targeting the key links of viral replication have become the core research directions for safeguarding the health of children and maintaining public health security.

At present, the mechanism of enterovirus infection, pathological characteristics, and the development of vaccines and antiviral drugs remain the focuses of research on HFMD. Advances in pediatric medicine and vaccine deployment are altering the morbidity and mortality patterns of HFMD in children (14). Nevertheless, many questions remain unanswered regarding the epidemiology, etiology, and pathogenesis of HFMD in children. To better characterize the clinical features and causes of death among children with severe HFMD, a retrospective analysis was conducted on 31 fatal cases managed in the pediatric intensive care units (PICUs) of Children's Hospital Affiliated to Zhengzhou University.

## 2 Materials and methods

### 2.1 Study population and sample collection

This retrospective study analyzed the clinical data of children with HFMD who were admitted to the PICUs of Henan Children's Hospital and died between January 2014 and December 2019. This study was approved by the Ethics Committee of Henan Children's Hospital. Informed consent was obtained from the guardian of each participant.

Inclusion criteria: (1). age less than 18 years; (2). met the diagnostic criteria of the Diagnosis and Treatment Guideline on hand-foot-mouth disease (2010) (15): ① clinical manifestations of HFMD, rash on the hands, feet, mouth, buttocks, and so on; ② positive nucleic acid test specific for enterovirus (CV-A16, EV-A71 and so on); (3). fatal outcome.

Exclusion criteria: (1). insufficient clinical data; (2). the absence of informed consent from the guardian.

### 2.2 Clinical data collection

Clinical data included the following: (1). baseline characteristics: sex, age, hospital stay, mechanical ventilation time, course of disease, and critical illness time point; (2). clinical manifestations: fever, rash cough, vomiting, capillary refilling time (CRT), nervous system manifestations, complications, and others; (3). etiological test results and time distribution of fatal cases; (4). laboratory results and coinfection; (5). imaging examinations.

Clinical samples (fecal and anal swabs, blood, throat swabs, sputum) were collected by dedicated nurses within 24 h, and etiological and laboratory tests were performed. When nervous system, respiratory system, or circulatory system involvement was suspected in the child, appropriate imaging (head MRI or head CT, chest x-ray or chest CT, echocardiography and electrocardiography) were performed immediately as clinically indicated.

### 2.3 Enterovirus typing methods

Fecal samples and anal swab samples were obtained from 31 patients. Samples were transported in cold storage and frozen at −20℃ and below. The samples were sent to the Henan Provincial Center for Disease Control and Prevention for testing. Viral RNA was extracted from the supernatants using QIAamp Viral RNA Mini Kit (Qiagen, Hilden, Germany). The Quantitative one-step RT-PCR was carried out using the Quant One Step RT-PCR Kit (Tiangen Biotech, Beijing, China). Detection of EV71, CA16, and pan-enterovirus RNA was performed using a real-time RT-PCR kit (Jinhao Pharmaceutical, Beijing, China). EV71 and CA16 nucleic acid detection primers and human enterovirus (EV) nucleic acid detection universal primers, reaction system and amplification conditions refer to the detection technology scheme of Guideline on preventions and controls of hand-foot-mouth disease of 2008 (16). See Supplementary S1 for details.

### 2.4 Statistical analysis

SPSS 27.0 statistical software was used for data analysis. Quantitative data of normal distribution are expressed as the mean ± standard deviation ($\bar{\chi }$ ± S), while non-normally distributed data are expressed as the median (P25, P75). The classification data are expressed as percentages (*n*, %).

## 3 Results

### 3.1 Baseline characteristics

From January 2014 to December 2019, 31 children with HFMD who were treated in the PICUs of our hospital died. None of the subjects had underlying disease. The male to female ratio was 1.58:1.00. The median age was 19.00 (12.00, 32.00) months, ranging from 9 months to 47 months. Hospital stay, critical illness time point, mechanical ventilation time and disease duration are shown in Table 1.

**Table 1:** Baseline characteristics and clinical manifestations.

Factors	Number
Baseline characteristics	
Sex (male/female, *n*)	19/12
Age, month, M (Q1, Q3)	19 (12, 32)
<12 months (*n*, %)	6, 19.35%
12∼<36 months (*n*, %)	18, 58.06%
≥36 month (*n*, %)	7, 22.58%
hospital stay, day, M (Q1, Q3)	1.25 (0.35, 6.00)
Mechanical ventilation time, day, M (Q1, Q3)	1.15 (0.48, 6.00)
Disease duration, day, M (Q1, Q3)	4.50 (3.50, 8.00)
Critical illness time point, day, M (Q1, Q3)	3.00 (2.00, 4.00)
Clinical manifestations	
Fever	31, 100.00%
Maximum temperature, °C, ($\bar{\chi }$±s)	(39.27±0.80)
Duration, days, M (Q1, Q3)	4.50 (3.00, 5.50)
Cough (*n*, %)	3, 9.68%
Vomiting (*n*, %)	21, 67.74%
Hypodynamia (*n*, %)	6, 19.35%
Hypotonia (*n*, %)	16, 51.61%
Convulsion (*n*, %)	5, 16.13%
Limb shaking (*n*, %)	22, 70.97%
Disturbance of consciousness (*n*, %)	30, 96.77%
Hypersomnia (*n*, %)	9, 29.03%
Light coma (*n*, %)	15, 48.39%
Deep coma (*n*, %)	6, 19.35%
Abnormal heart rate (*n*, %)	29, 93.55%
1∼1.5ULN (*n*, %)	15, 48.39%
1.5∼2.0ULN (*n*, %)	12, 38.71%
CRT≥3 s (*n*, %)	24, 77.42%
Shock index	
0.5∼1.0 (*n*, %)	2, 6.45%
1.0∼1.5 (*n*, %)	10, 32.26%
1.5∼2.0 (*n*, %)	10, 32.26%
>2.0 (*n*, %)	9, 29.03%
Coagulation disorder dysfunction (*n*, %)	6, 19.35%

Note. ULN: upper limit of normal, see Supplementary S1 for details.

### 3.2 Clinical manifestations

All the 31 children with HFMD had fever and rash, with an avege maximum temperature of 39.27℃. There were 3 cases (9.68%) with low fever (37.5–38.0℃), 5 cases (16.13%) with moderate fever (38.1–38.9℃) and 23 cases (74.19%) with high fever (39.0–40.9℃). The median duration of fever was 4.50 (3.00, 5.50) days.

All patients had dyspnea and required mechanical ventilation. Additional common manifestations included disturbance of consciousness, abnormal heart rate, CRT ≥ 3 s, limb tremors, vomiting and hypotonia. Among the 31 cases, 30 cases (96.77%) experienced disturbance of consciousness, which was manifested as hypersomnia, light coma and deep coma. The diagnostic criteria for disturbance of consciousness are detailed in Supplementary S2. Light coma was the most frequent, with 15 cases (48.39%). Abnormal heart rate was observed in 29 children (48.39%), comprising abnormal rapid heart rate in 27 children and abnormal slow heart rate in 2 children. The rapid heart rate was mostly in the range of 1–1.5 ULN, occurring in 15 cases (48.39%) (Table 1, Supplementary S3). The shock index is calculated by dividing the heart rate by the systolic blood pressure. In this study, we found that 29 cases (93.55%) exhibited a shock index exceeding 1.00, while 9 cases (29.03%) demonstrated a shock index greater than 2.0. The clinical manifestations are summarized in Table 1.

All children with fatal HFMD presented with neurological complications, including 21 cases of NPE, 17 cases of brainstem encephalitis, 12 cases of encephalitis, 3 cases of cerebral hernia (tentorial hernia, cerebellotonsillar hernia), 3 cases of central diabetes insipidus, and 2 cases of encephalomyelitis. Additionally, there are many complications such as stress ulcers, liver injury, pneumonia, bronchitis, pneumothorax, myocardial injury, fulminant myocarditis and kidney injury. Complications can involve multiple systems at the same time, and it is not uncommon for multiple complications to occur in the same system. Details of the complications are shown in Table 1.

### 3.3 Etiological test results and time distribution of fatal cases

The results of the etiological examination revealed that EV-A71 was the main pathogen of fatal HFMD cases. There were 24 cases positive for EV-A71, 3 cases positive for pan-enterovirus, 1 case positive for CV-A16, and 3 cases positive for both EV-A71 and pan-nterovirus.

**Figure 1:**
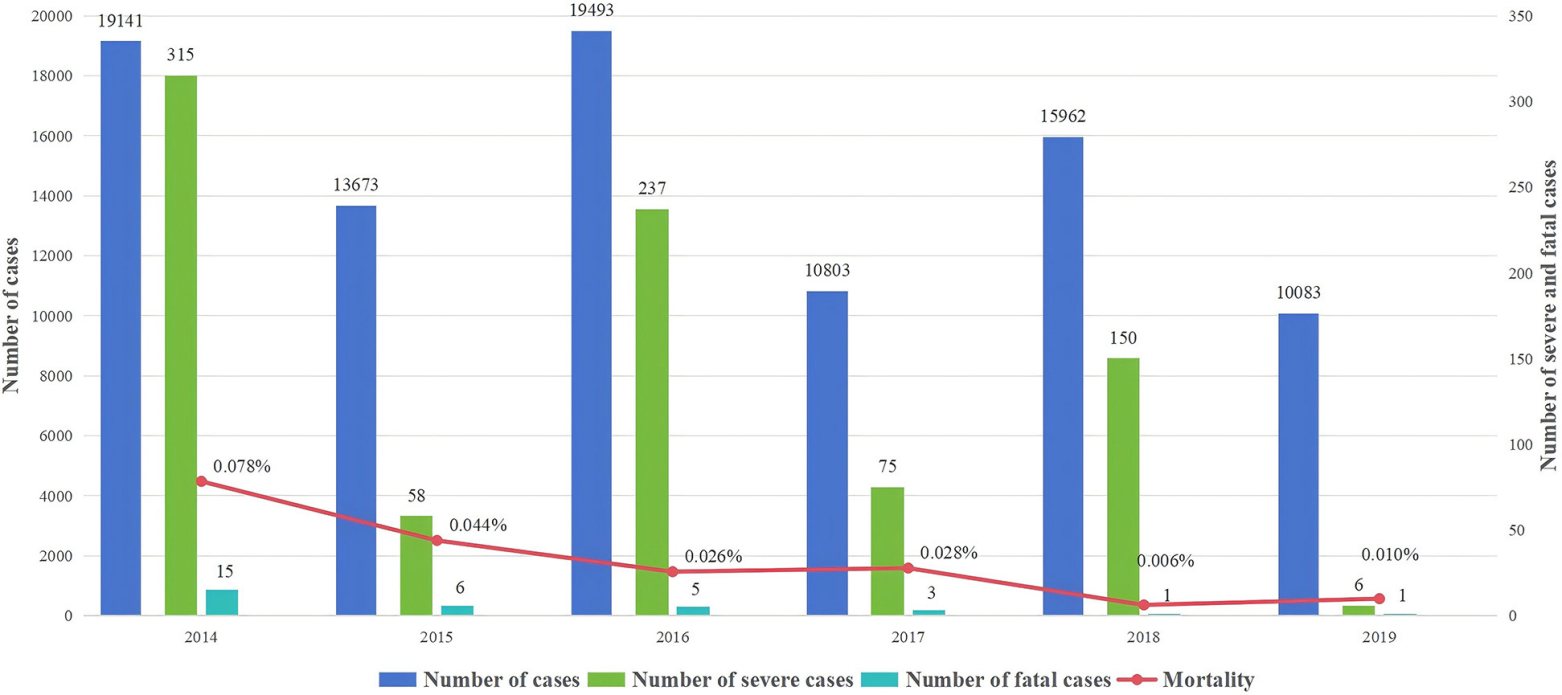
The annual case number of cases, severe cases, fatal cases and mortality of HFMD in Children's hospital affiliated to Zhengzhou university from 2014 to 2019.

Henan is one of the most serious HFMD epidemic provinces in China, and Children's Hospital Affiliated to Zhengzhou University is the largest tertiary level A pediatric hospital in Henan and a sentinel hospital for surveillance of HFMD and romotion and implementation centers of prevention and treatment for HFMD designated by the Chinese state. Based on this background, our previous studies have conducted relevant studies on the incidence of HFMD in children in Henan (17). Furthermore, this study counted the number of deaths of children with HFMD treated at the Children's Hospital Affiliated to Zhengzhou University from 2014 to 2019 and plotted the time distribution (Figure 1). The results showed that the number of HFMD cases, severe cases, fatal cases and mortality showed a decreasing trend. The number of children with HFMD died in Children's Hospital Affiliated to Zhengzhou University from 2014 to 2019 was 31. The number of deaths peaked at 15 in 2014 and subsequently declined. Moreover, there were no fatal HFMD cases occurred at this hospital between 2020 and 2024.

### 3.4 Laboratory results and coinfection

Elevated white blood cell (WBC) counts were observed in all 31 patients with a median of 14.34 (10.44, 20.00) 10^−9^/L. The majority of white blood cells were neutrophils, and the average platelet count was 404.71 10^−9^/L. C-reactive protein (CRP) in 24 cases (77.42%) was within the normal range, and CRP was elevated in 7 cases. The highest CRP was 53.09 mg/L. Serum procalcitonin (PCT) was elevated in 21 patients, with a median of 1.06 ng/ml. Liver enzymes were abnormally elevated in 23 cases. The maximum values of alanine aminotransferase (ALT) and aspartate aminotransferase (AST) levels were 4276.90 U/L and 7500.00 U/L, respectively. Myocardial enzymes were elevated in 28 cases. The highest values of lactate dehydrogenase (LDH), cardiac troponin T (cTnT) and B-type natriuretic peptide (BNP) were 7376.20 U/L, 8.73 ng/ml and 35000.00 pg/ml, respectively. The serum levels of neuron-specific enolase (NSE) and S100 were elevated. Blood gas analysis identified 13 cases with abnormal PaO2/FiO2 ratios, with a median value of 310.00. The values of PaO_2_/FiO_2_ ranged from 200.00 to 300.00 in 9 cases, 100.00 to 200.00 in 3 cases, and <100.00 in 1 case. The laboratory results are shown in Table 2.

**Table 2:** Laboratory results.

Factors	Number
WBC, 10^−9^/L	14.34 (10.44, 20.00)
Platelet, 10^−9^/L	404.71±186.85
Neutrophil percentage, %	72.54±8.99
CRP, mg/L	3.55 (0.80, 9.83)
PCT, ng/ml	1.06 (0.16, 2.55)
ALT, U/L	29.40 (17.75, 65.88)
AST, U/L	68.50 (42.00, 167.73)
AGR	1.39±0.40
Creatinine, umol/L	35.00 (20.75, 55.50)
LDH, U/L	345.80 (300.10, 777.20)
cTnT, ng/mL	0.38 (0.02, 0.92)
BNP, pg/ml	4989.00 (1603.50, 9544.50)
NSE, ng/ml	31.64 (23.55, 54.42)
S100, ug/L	0.31 (0.17, 0.70)
PaO2/FiO2	310.00 (223.00, 440.00)
Lactate, mmol/L	2.20 (1.40, 4.70)

**Figure 2:**
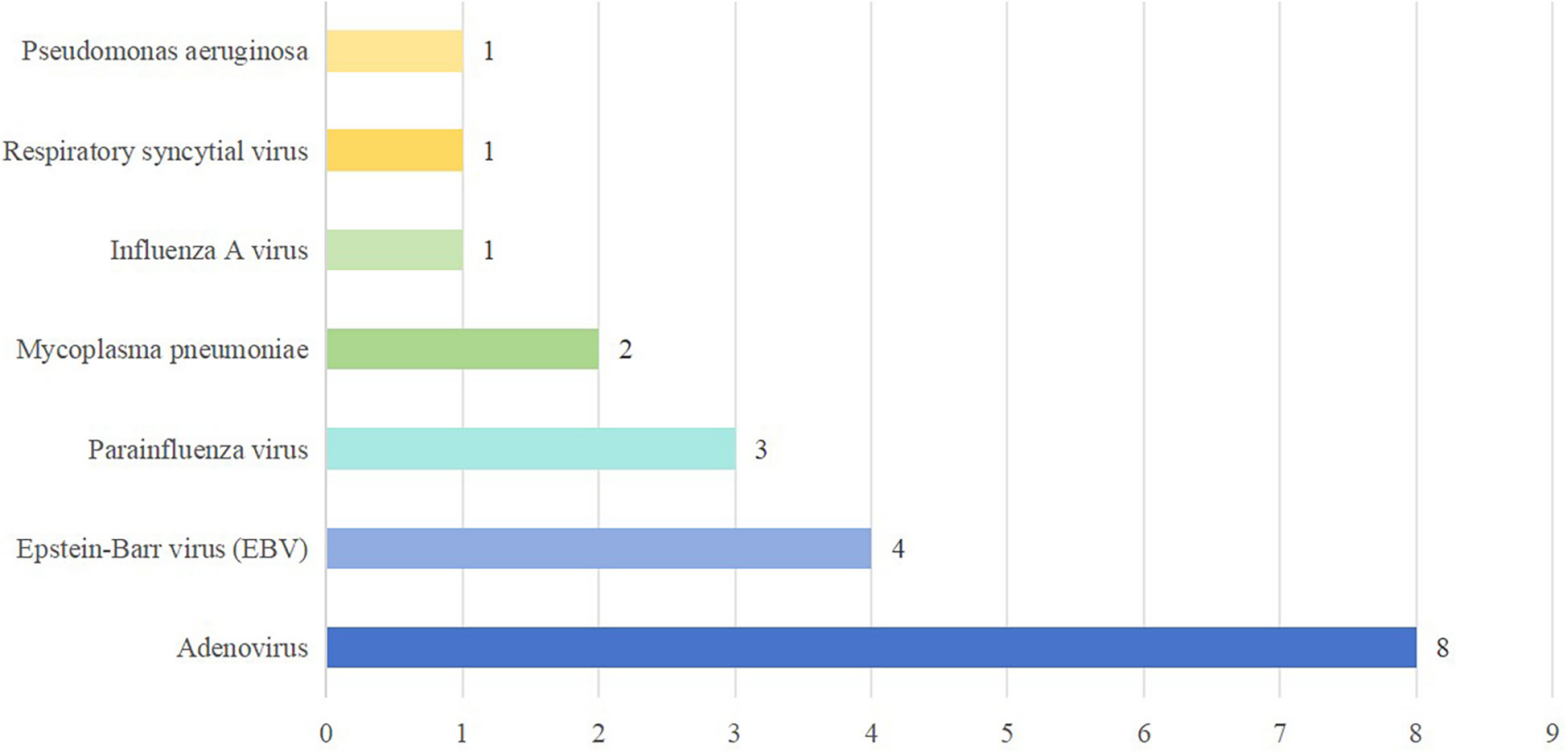
Coinfection in fatal cases of HFMD. Patients can be coinfected with multiple pathogens.

Etiological testing identified coinfection in 16 (51.61%) of the 31 fatal cases, predominantly viral. The top three coinfected pathogens were adenovirus, Epstein–Barr virus (EBV) and parainfluenza virus. Coinfection with multiple pathogens is not uncommon. The details of coinfection in fatal cases of HFMD are shown in Figure 2.

With guardian informed consent, cerebrospinal fluid (CSF) was performed in 8 patients. Routine and biochemical CSF findings were consistent with viral neurological infection. Neuron-specific enolase levels were measured in the CSF of 5 patients with informed consent of the guardian, and all were elevated (mean: 41.44 ng/ml), indicating nervous system damage.

### 3.5 Imaging examination

**Figure 3:**
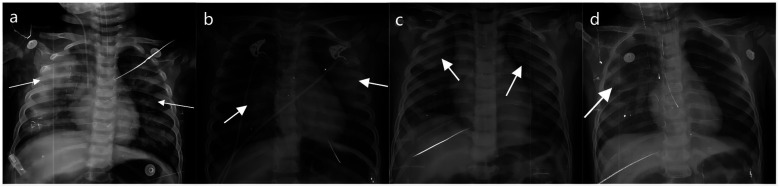
The chest radiograph of NPE in fatal cases of HFMD. **(a–d)** Shows different degrees of NPE in children with HFMD. The arrow points to the lesion.

Pulmonary hemorrhage may occur in the later stage of NPE, and the chest radiograph shows decreased translucency, ground glass-like changes and widely distributed patchiness in both lung fields (Figure 3).

**Figure 4:**
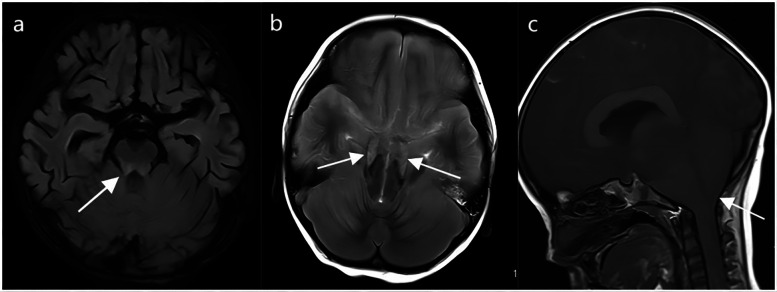
The cranial MRI in fatal cases of HFMD. **(a)** Abnormal brain stem signals from brain stem encephalitis in HFMD. **(b)** Tentorial hernia in HFMD. **(c)** Cerebellotonsillar hernia in HFMD. The arrow points to the lesion.

Cranial MRI of children who died from brainstem encephalitis showed patchy T1 and T2 signals in the brainstem (Figure 4a). Figures 4b,c illustrate cranial MRI findings of tentorial hernia and cerebellotonsillar hernia, respectively.

### 3.6 Causes of death

Among the 31 fatal cases, 24 cases died of NPE, and 18 of them had severe pulmonary hemorrhage. The hospital stay, mechanical ventilation time and disease duration were 0.06–6.00 days, 0.08–6.00 days and 1.00–10.00 days, respectively. The time of onset of critical illness was 3.00 ± 1.18 days, and 21 patients died within 7 days of symptom onset.

Six cases died of brainstem encephalitis. The hospital stay, mechanical ventilation time and disease duration were 7.00–125.00 days, 7.00–62.00 days and 9.50–174.00 days, respectively. The time of onset of critical illness was 2.92 ± 2.01 days. These children have brain stem damage and no spontaneous breathing. The patient could not maintain normal respiratory function after stopping mechanical ventilation. These children died quickly after their parents abandoned treatment and withdrew from mechanical ventilation. The median age of the children who died from NPE was 17.50 (12.00, 31.50) months, whereas the median age of the children who died from brainstem encephalitis was 25.00 (16.50, 37.75) months.

In addition, a 28-month-old child died of cerebral hernia, with a disease duration of 4.50 days, and his condition became critical on the third day of the disease.

## 4 Discussion

HFMD remains a serious threat to children's health as a common infectious disease in China. Although often considered a mild and self-limiting disease usually resolving within 7 days, a subset of children with HFMD progress to severe cases with high mortality. HFMD, a viral infectious disease, lacks specific treatment (8). Early identification of the clinical manifestations of fatal cases is a key measure to reduce the mortality of severe HFMD. Our study analyzed the comprehensive clinical features of children who died from HFMD.

The susceptibility and severity of HFMD are age-related (18). Children under 5 years of age constitute the most affected group, accounting for more than 90% of cases (2). Furthermore, children under 2 years of age are at higher risk for serious complications and mortality; and 84.02% of deaths occurred in this group (2). Our findings align with these findings. All cases were children under 5 years old with an average age of 23.13 months. The highest proportion of cases was 58.06% from 12 months to 36 months, and 24 cases (77.42%) were under 2 years old. Increased susceptibility in young children may relate to cluster epidemics and immature immune protection. Epidemiological data show that morbidity and mortality are greater in younger children (19). The median hospital stay was 1.25 (0.35, 6.00) days. The median critical illness time point, mechanical ventilation time and disease duration were 3.00 (2.00, 4.00) days, 1.15 (0.48, 6.00) days and 4.50 (3.50, 8.00) days, respectively. These results suggest that the disease progresses rapidly and that the disease duration is short. Most fatalities occurred within one week. In addition, none of the fatal cases had underlying disease.

Timely and accurate identification of risk signs and symptoms during disease progression is particularly important for the diagnosis and treatment of severe HFMD. Identification and proper treatment can effectively reduce the mortality (4). Therefore, our study provided a more detailed description of the signs and symptoms. All patients had clinical manifestations of fever, rash, and dyspnea. Relevant studies have shown that a fever exceeding 39°C for more than 3 days is closely associated with severe or fatal HFMD (4, 20). In our study, high fever was the main form of fever, and 83.87% of the children had a history of fever for more than 3 days. In addition, disturbance of consciousness, abnormal heart rate, CRT ≥ 3 s, limb tremors, vomiting and hypotonia were the common clinical manifestations. Disturbance of consciousness, limb tremors, vomiting and hypotonia all indicate nervous system involvement and poor prognosis. These clinical manifestations indicate more extensive or severe neurological damage.

EV-A71 and CV-A16 are the most commonly reported causative agents of HFMD. Other enteroviruses, such as CV-A6 and CV-A10, currently account for a significant proportion of children with HFMD, and their epidemiological influence is expanding (21, 22). EV-A71 infection is the leading cause of death and an independent risk factor for death from severe HFMD (6, 23). Consistently, the most common pathogen in our fatal cases was EV-A71, with a positive rate of 87.10%. EV-A71 is one of the most important neurotropic viruses, and the mechanism of its induction of neurological deficits remains unclear. EV-A71 infection of intestinal epithelial cells progresses to viremia, which may invade the central nervous system later by disrupting the blood-brain barrier and retrograde axonal transport (6). Human SCARB2 (24) and CXCL1-CXCR2 signaling pathway are involved in the neurological function impairment of EV-A71, and CXCL1-CXCR2 signaling pathway has been confirmed to be related to the severity of neurological complications (25).

In September 2016, Henan Province officially offered EV-71 vaccination to children. With the development and application of the EV-A71 vaccine, the morbidity and mortality patterns of HFMD have changed (14). Our hospital is the national children's regional medical center, which undertakes the diagnosis and treatment of most critically ill children in Henan Province and some of the neighboring provinces, which can better reflect the regional disease epidemic situation. Therefore, we analyzed the morbidity and mortality of children with HFMD treated in our hospital from 2014 to 2019. The results revealed that with the continuous improvement of diagnosis and treatment experience and the promotion of vaccines, the acute mortality of children with severe HFMD has gradually decreased. However, the potential for possible short-term or long-term sequelae remains a concern. The long-term sequelae of severe HFMD primarily affect preschoolers under 5 years of age, a critical stage in their growth and development. In recent years, the molecular epidemiology of HFMD pathogens has changed, potentially leading to outbreaks, and the development of pan-enterovirus vaccines is a possible solution (26).

Children with HFMD may have increased WBC counts, elevated of liver or myocardial enzymes, and abnormal blood gas (4). Our laboratory results showed increased WBC counts dominated by neutrophils and mostly elevated PCT. Abnormal liver or myocardial enzymes were common. Thirteen patients had abnormal pulmonary function, with significantly reduced PaO2/FiO2 ratios. Several studies have shown that WBC, BNP, and PCT can be used as early biomarkers of severe or fatal HFMD and are positively correlated with disease severity (27–29). Severe HFMD can lead to rapid cardiopulmonary failure, and BNP has been widely used in the diagnosis of cardiopulmonary failure and the prognosis of cardiovascular diseases (27, 28).

Our study revealed that more than half of the pediatric patients exhibited coinfection with enteroviruses and other pathogens, with adenoviruses being the most common. Relevant studies have shown that both adenovirus and influenza A virus can cause serious damage to the central nervous system (30–32). However, potential interactions between these viruses and enteroviruses, especially EV-A71, in promoting severe central nervous system complications remain unknown. One patient had *Pseudomonas aeruginosa* coinfection, likely related to long-term mechanical ventilation. He was mechanically ventilated for 62 h and was hospitalized for 125 days. The correlation between multipathogen coinfection and the severity of HFMD needs further study. Coinfection is closely related to the severity and complications of the disease, and the coinfection of different enteroviruses may enhance the pathogenic effect of the disease (33). Indeed, there have been reports describing the synergistic effects among coinfected pathogens that can lead to pathogenic mechanisms (34), and also promote the emergence of atypical clinical manifestations and disease severity (35–38). Therefore, monitoring coinfection is crucial for understanding atypical presentations and disease severity in children with HFMD.

In this study, NPE and brainstem encephalitis were the main causes of death in children with HFMD, consistent with prior studies (4, 9). Enterovirus invasion of the central nervous system can cause brain edema and injury, further inducing a systemic inflammatory response and immune damage, and eventually leading to NPE and other serious complications (9, 10, 39). NPE is one of the most serious central nervous system complications and a leading cause of rapid death in children with severe HFMD, and its development is closely linked to central nervous system inflammation and cytokine storms (6, 14, 40). NPE progresses rapidly, carries high mortality, and survivors face significant neurological sequelae risk (9). Lijun Peng pointed out that drowsiness, vomiting, tachycardia, hypertension, respiratory rhythm change, and limb tremor were risk factors for NPE in children with severe HFMD, and approximately 90% of patients died within 12 h (10). NPE progresses rapidly and has a very high mortality rate. Even those who survive are at great risk of neurological sequelae (9). Previous studies have shown that NSE and S100 are highly expressed in neurons and can be used as sensitive markers of brain injury (41, 42). These markers are significantly upregulated in inflammatory processes such as trauma, infection, heat and stress and play a role in inflammation regulation (43, 44). We found that NSE and S100 were elevated in children with HFMD. It is urgent for pediatricians to identify these potential risk factors early and take countermeasures to reduce mortality in children with severe HFMD.

There are several limitations in this study. First, the sample size of this study is small, and the comparison of children who died from neurogenic pulmonary edema vs. those with brainstem encephalitis. It is still necessary to collect relevant cases for basic comparative analyses. Second, the quality of the historical imaging data (2014–2019) utilized in this study is constrained by the acquisition parameters and storage technologies available at the time. Consequently, these images possess inherent limitations in resolution that could not be retrospectively enhanced. Third, the coinfection in children with HFMD awaits further research. Further studies are needed to clarify the potential synergistic mechanisms among pathogens and their impact on the severity of the disease. Forth, some clinical indexes in the fatal cases may not be detected, which affects further study of the pathogenesis of these fatal cases. Fifth, the serology markers of hepatitis, e.g., HBV surface antigens, were missing, which may be helpful for explaining the elevated AST and ALT levels in our cohort (45–47).

## 5 Conclusions

Fatal HFMD predominantly affects children, especially those under 36 months of age. Mortality rate of HFMD has declined in recent years. EV-A71 is the primary pathogen of death from HFMD. Neurological complications were predominant, and the main causes of death were NPE and brainstem encephalitis. Children with brainstem encephalitis had longer hospital stay and mechanical ventilation dependence. In comparison, NPE and pulmonary hemorrhage progressed more rapidly, with shorter disease duration, and these children died within 7 days of symptom onset.

## Data Availability

The raw data supporting the conclusions of this article will be made available by the authors, without undue reservation.
