# Supplementary material for: Clinical characteristics of fatal cases of hand, foot and mouth disease in children
Source: Front Pediatr. 2025 Jul 17;13:1522164. doi: 10.3389/fped.2025.1522164 (PMC12310577; doi:10.3389/fped.2025.1522164)
Supplement: Supplementary file 1 [file Datasheet1.docx]

1. **RT-PCR amplification**

(1) Primer sequence synthesis

1) Human enterovirus (including EV71, CA16) nucleic acid detection universal primer sequence:

PE2 (upstream) : 5 '-TCC GGC CCC TGA ATG CGG CTA ATC C-3'

PE1 (downstream) : 5 '-ACA CGG ACA CCC AAA GTA GTC GGT CC-3'

2) EV71 nucleic acid detection primer sequence:

EV71-S (upstream) : 5 '-GCA GCC CAA AAG AAC TTC AC-3'

EV71-A (downstream) : 5 '-ATT TCA GCA GCT TGG AGT GC-3'

3) Primer sequence for CA16 nucleic acid detection:

Cox A16-S (upstream) : 5 '-ATT GGT GCT CCC ACT ACA GC-3'

Cox A16-A (downstream); 5`-TCA GTG TTG GCA GCT GTA GG-3`

1. **RT-PCR experimental results explanation table**

| RT-PCR results | Identification result |
| --- | --- |
| All primers(－) | Non-Enterovirus |
| EV(＋), EV71(－), CA16(－) | Enterovirus universal |
| EV(＋), EV71(＋), CA16(－) | EV-A71 |
| EV(＋), EV71(－), CA16(＋) | CV-A16 |
