# Supplementary material for: Clinical characteristics of fatal cases of hand, foot and mouth disease in children
Source: Front Pediatr. 2025 Jul 17;13:1522164. doi: 10.3389/fped.2025.1522164 (PMC12310577; doi:10.3389/fped.2025.1522164)
Supplement: Supplementary file 2 [file Datasheet2.docx]

The degree of disturbance of consciousness(1)

| degree | Response to pain | wake-up test | Involuntary movements | tendon reflex | pupillary light reflex | vital sign |
| --- | --- | --- | --- | --- | --- | --- |
| hypersomnia | obvious | stimulus | ＋ | ＋ | ＋ | stable |
| lethargy | slow response | Strong stimulus | ＋ | ＋ | ＋ | stable |
| light coma | exist | － | ＋ | ＋ | ＋ | stable |
| deep coma | Strong stimulus | － | rarely | － | slow response | Mildly unstable |

1. Zhenjiang B, Ying L. Emergency diagnosis and management of coma in children2018. 1376-81 p.
