# Supplementary material for: Clinical characteristics of fatal cases of hand, foot and mouth disease in children
Source: Front Pediatr. 2025 Jul 17;13:1522164. doi: 10.3389/fped.2025.1522164 (PMC12310577; doi:10.3389/fped.2025.1522164)
Supplement: Supplementary file 3 [file Datasheet3.docx]

| Age | Heart rate (bpm) |
| --- | --- |
| 0-28 days | 120-140 |
| <1 year | 110-130 |
| 1-3 years | 100-120 |
| 4-7 years | 80-100 |
| 8-14 years | 70-90 |

The above data refer to the Pediatrics, 9rd edn(1).

1. Ren X, Zhang A. Pediatrics, 9rd edn: Beijing: People's Medical Publishing House; 2020.
